# Impact of the value driven outcomes program among cataract surgery patients in Singapore: an interrupted time series analysis

**DOI:** 10.1186/s12913-023-09427-2

**Published:** 2023-05-15

**Authors:** Jian Ming Hoong, Brent Gibbons, Benedict Loh, Clement Tan, Cynthia Chen

**Affiliations:** 1 Saw Swee Hock School of Public Health, National University of Singapore, 12 Science Drive 2, #09-01T, Singapore, 117549 Singapore; 2 Ng Teng Fong General Hospital, National University Health System, Singapore, 609606 Singapore; 3 National University Hospital, National University Health System, Singapore, 119074 Singapore; 4 Schaeffer Center, University of Southern California, Los Angeles, CA 90089 USA

**Keywords:** Value-driven outcome, Cataract surgery, Healthcare value, Cost-effectiveness

## Abstract

**Background:**

Healthcare cost is increasing rapidly in Singapore. Moving towards a value-based healthcare framework enables a sustainable health system. The National University Hospital (NUH) implemented the Value Driven Outcome (VDO) Program for cataract surgery due to its high volume and cost variability. We aimed to evaluate the association between VDO program implementation and costs and quality outcomes for cataract surgery in NUH.

**Methods:**

We conducted an interrupted time-series analysis for cataract surgery episodes between January 2015 and December 2018. Using segmented linear regression models, we estimate the changes in levels and trends of cost and quality outcomes post-program implementation. We adjusted for autoregression and various confounders.

**Results:**

Following VDO program implementation, the total cost of cataract surgery had a significantly decreased by $327.23 (95% CI: -$421.04 to -$233.43; *p* < 0.01) and the trend significantly decreased by $13.75 per month (95% CI: -$23.19 to -$4.30 per month; *p* < 0.01). There was a small improvement in the combined quality outcome score (0.028, 95% CI: 0.016 to 0.040; *p* < 0.01), but the trend remained unchanged.

**Conclusion:**

The VDO program was associated with a reduction in cost without compromising on quality outcomes. The program provides a structured methodology to measure performances, and through these data, initiatives were implemented to improve value. There are benefits to providing a data reporting system to physicians to understand actual care costs and quality outcomes achieved by individual patients with defined clinical conditions.

## Background

The cost of healthcare is increasing rapidly in Singapore. Since 2010, healthcare spending had almost doubled, from $11 billion to $21 billion in 2016. In the same period, the government health expenditure, excluding out-of-pocket, Medisave, and private payments, increased 2.4 times from $3.9 billion to $9.3 billion [1]. Healthcare spending is expected to continue increasing with Singapore’s rapidly ageing population and rising life expectancy. The rising trend of chronic disease further exacerbates the healthcare spending concerns [2]. This exponential increase in national healthcare expenditures is unsustainable, and the healthcare system needs to adapt to mitigate the rising health expenditure. Reforming healthcare systems based on value outcomes has the potential to improve performance. As part of the Beyond Healthcare 2020 plan by the Singapore’s Ministry of Health [3] to enable a more sustainable health system, one of the key paradigm shifts includes “beyond quality to value”. This shift recognises the importance of improving healthcare value for patients, which can be defined as the quality outcomes achieved per dollar spent.

The University of Utah had deployed a quality-of-care data model known as the Value Driven Outcomes (VDO) tool in 2012 [4, 5]. It was created to improve healthcare value, with an aim to help providers value the trade-off between the cost, quality outcomes and patient’s experience of their medical condition in a timely manner. This data can help to improve the organisation of care, allowing providers to redesign care [6]. By adopting the well-established business practice of “If you can’t measure it, you can’t improve it”, the university seeks to improve healthcare value. Therefore, the VDO program aims to provide cost and quality outcome measurements through scorecards and dashboards to improve system processes and patient outcomes.

The National University Health System (NUHS) VDO program adopted similar strategies through the measurement of quality outcomes and cost. A variety of outcome indicators were selected by specialist physicians to measure condition-specific treatments, for example, cataract surgery. Quality outcomes can include process outcomes (e.g. length of stay), health and clinical outcomes (e.g. re-admission within 30 days) and patient-reported outcome measures (PROMs) (e.g. patient experience) [7]. These outcome measures are summed into a quality outcome score. Cost is defined as the total cost to deliver the treatment over the full episode of care for a specific medical condition. One of the medical conditions identified by NUHS to be suitable for the VDO program is cataract surgery. It was identified due to its significant disease burden and high volume (more than 4000 cases are treated annually in NUH). It is also one of the top ten surgeries by volume done in public hospitals in Singapore [8], and the average total cost and the cost variation is high between similar cases [8]. Furthermore, the Projection of Eye Disease Burden study in Singapore [9] reported that the prevalence of cataract would increase by 81% to 1.33 million by 2040. Therefore, the goal of increasing value for patients undergoing cataract surgery ensures that the healthcare system remains effective and economically sustainable as the prevalence of people with cataract and needing a cataract surgery increase. In this study, we aimed to evaluate if the implementation of the VDO program improves value (i.e., reduces cost and improves quality outcomes) for patients undergoing cataract surgery in NUH. This paper (1) identifies overall cataract surgery and care costs across the health care system, and (2) allows stakeholders to understand the importance of value-based healthcare which ultimately supports value improvement initiatives for selected conditions.

## Methods

Ethics has been reviewed and approved by the National Healthcare Group’s Domain Specific Review Board (DSRB) (Ref: 2018/00626). A list of cataract surgery episode in NUH, its associated cost and quality outcomes from January 2015 to December 2018 was obtained from the NUH Academic Information Office and Finance Department.

### Cost indicators

Cost data related to each cataract surgery encounter were obtained from the NUH Finance department. These costs include time spent by surgeon and anaesthetist depending on the length of surgery, use of OT equipment, investigative equipment and the actual procurement cost of medication and supplies. For each episode, we included Accident and Emergency, Consultation, Consumables, Daily Treatment Fee, Dental Services, Doctor’s Fee, Investigations, Medications, Non-Treatment Services, Renal Dialysis, Room Charges, Surgery Services, Therapy Services and Treatment Services. The total cost from these services were summed and adjusted to 2018 Singapore Dollars.

### Quality outcome indicators

Outcome indicators were obtained from the NUH Academic Information Office. These indicators were selected by specialist physicians for the cataract surgery VDO track prior to implementation. There are ten outcome indicators measured, and these include:

1. No day surgery turned inpatient
2. No vitreous loss
3. No endophthalmitis
4. No zonulysis
5. No corneal decompensation
6. No other eye-related post-operative complication
7. No other postoperative complication
8. No unscheduled return to operating theatre (OT) in 90 days
9. No all-cause re-admission in 30 Days
10. Patient experience score of 8 or higher

These quality indicators were then summed into a quality outcome score with a maximum value of 10, where a high score indicates better quality outcomes.

Changes in cost and quality outcomes were assessed between a baseline period of January 1, 2015, to May 31, 2017, and an evaluation period of June 1, 2017, to December 31, 2018. A phase-in period between June 1, 2017 to August 31, 2017 was considered as part of a sensitivity analysis. The list contains 10,981 episodes of hospital stay with cataract surgery based on the Ministry of Health’s table of surgical procedures code. We excluded episodes that were primarily for non-eye related procedures (e.g., hip arthroplasty, limb amputation, etc.) but also had cataract surgery in the same episode (*n* = 20), and those who had multiple eye surgeries (*n* = 146) as the aim of this study was to evaluate the effect of the VDO program on cataract surgery alone. Overall, there were 10,815 episodes included in the evaluation.

### Statistical analysis

We conducted an interrupted time series (ITS) analysis, using a longitudinal quasi-experimental study design, to evaluate the association between the VDO program and the cost and quality outcomes of patients undergoing cataract surgery in NUH. ITS analyses are mainly used for evaluating natural experiments such as policy changes [10, 11]. Compared to other before-and-after analytical techniques such as the means comparison tests, ITS analyses account for pre-trends and reduces the possibility that the associations observed are due to the pre-intervention trends rather than the intervention. ITS analyses are also able to detect trend changes, which are useful when changes are expected to take place over a period of time [10, 11].

Our ITS analysis uses segmented generalised least square linear regression models to examine the mean total cost and overall quality outcome score. These are measures that the VDO program intend to improve. For every time series model, we included a pre-intervention period of 29 months to control for biases in level and trend at baseline, and a post-intervention period of 19 months to study the impacts of the program. Time periods were selected to coincide with the implementation of the VDO program in June 2017. We also evaluated each time series analysis for confounding by autoregression (autocorrelation and moving average) using the Durbin Watson Test [12] and the autocorrelation and partial autocorrelation function plot [13]. It was decided that if autoregression was identified, it would be modelled into the ITS models based on the lag value obtained. A likelihood ratio test was also conducted to ensure that adding the autoregression parameter will improve the model fit.

We report three nested models for each outcome. Model 1 is the unadjusted model, Model 2 adjusts for demographic variables including age, gender and subsidy status, and Model 3 adjusts for variables in Model 2 as well as clinical variables such as comorbidities using Charlson Comorbidity Index (CCI) and the fitness of patients before surgery using American Society of Anaesthesiologists’ (ASA) Score. Gender was included as prior research suggested differing quality outcomes for cataract surgery between men and women [14, 15] and ASA score was included to adjust for differences in patients’ fitness before the surgery [16]. We used Stata 16 and R software for all statistical analyses. All hypothesis tests were performed using 2-sided α = 0.05.

## Results

Comparing patients’ characteristics before and after VDO program implementation (Table 1), patients tend to be younger (67.8 vs 69.5 years, *p* < 0.01), had higher comorbidity score (0.84 vs 0.68, *p* < 0.01), and had lower ASA score (2.14 vs 2.17, *p* < 0.01) post implementation. A summary of the quality outcome measures, which was described earlier, during the pre- and post-implementation periods were presented in Table 2. The proportion of patients with zonulysis postoperatively decreased (2.63 vs 1.83%, *p* < 0.01), and patients with lower experience score (lower than 8) decreased as well (1.88 vs 0.49, *p* < 0.01). Overall, patients after the implementation of the VDO program tended to have slightly better outcomes.

**Table 1:** Patient’s characteristics

Demographic	Before VDO Implementation (*n* = 5901)	After VDO Implementation (*n* = 4914)	*P*-value
**Age (years), Mean (SD)**	69.5 (10.1)	67.8 (9.61)	** < 0.01**^**a**^
**Gender, n (%)**			
Male	2,904 (49.2)	2,380 (48.4)	0.42^b^
Female	2,997 (50.8)	2,534 (51.6)	
**Race, n (%)**			
Chinese	4,386 (74.2)	3,627 (73.8)	0.05^b^
Malay	801 (13.6)	644 (13.1)	
Indian	428 (7.3)	346 (7.1)	
Others	286 (4.9)	297 (6.0)	
**CCI, Mean (SD)**	0.68 (0.56)	0.84 (0.48)	** < 0.01**^**a**^
**ASA Score, Mean (SD)**	2.17 (0.49)	2.14 (0.48)	** < 0.01**^**a**^
**Subsidy Status, n (%)**			
Subsidised	4,545 (77.0)	3,712 (75.5)	0.07^b^
Unsubsidised	1,356 (23.0)	1,202 (24.5)	

*CCI* Charlson Comorbidity Index, *ASA Score* American Society of Anaesthesiologists’ Score

^a^ Independent sample t-test

^b^ Chi-sq test

**Table 2:** Quality outcome measures

**Quality Outcome**	**Before VDO Implementation** Frequency (%) *n* = 5901	**After VDO Implementation** Frequency (%) *n* = 4914	***p*-value**
**Day surgery turned inpatient**	130 (2.20)	89 (1.81)	0.15^a^
**Vitreous loss**	20 (0.34)	23 (0.47)	0.29^a^
**Endophthalmitis**	0 (0)	0 (0)	-
**Zonulysis**	155 (2.63)	90 (1.83)	** < 0.01**^**a**^
**Corneal decompensation**	2 (0.03)	2 (0.04)	0.62^b^
**Other eye related post-operative complication**	18 (0.31)	13 (0.26)	0.70^a^
**Other post-operative complication**	192 (3.25)	122 (2.48)	**0.02**^**a**^
**Unscheduled return to operating theatre (OT) in 90 days**	41 (0.69)	30 (0.61)	0.59^a^
**All cause re-admission in 30 Days**	128 (2.17)	92 (1.87)	0.28^a^
**Patient experience score lower than 8**	111 (1.88)	24 (0.49)	** < 0.01**^**a**^

^a^ Chi-sq test

^b^ Fisher’s exact test

### Total cost

**Fig. 1:**
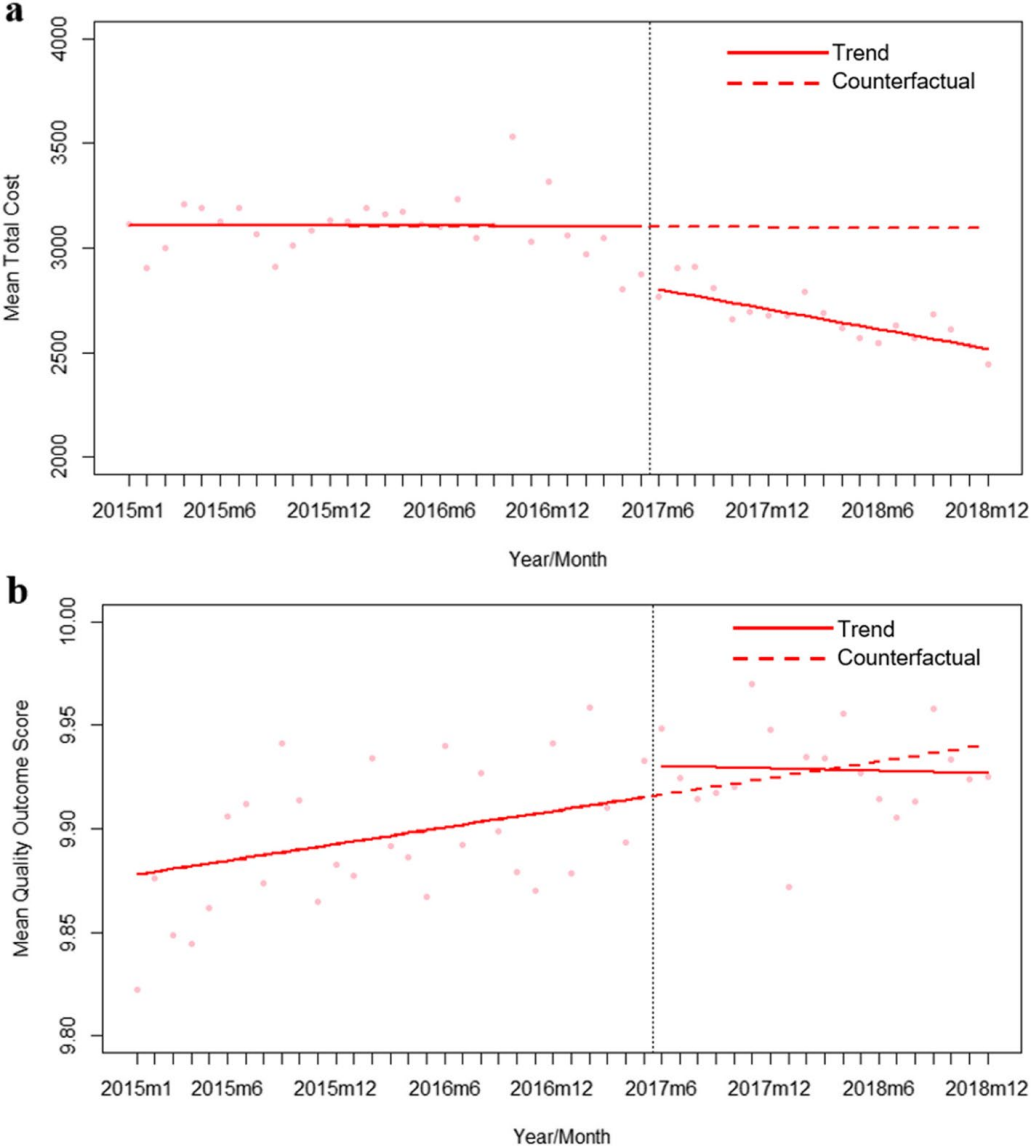
Interrupted time series of the mean total cost (**a**) and quality outcomes (**b**) of cataract surgery encounters by month from January 2015 to December 2018

Figure 1a showed the mean total cost of cataract surgery in NUH from January 2015 to December 2018. In the months prior to the VDO program, the mean costs for each month ranged from $2804 to $3534 and there was no significant change in cost over time. In Model 1 (unadjusted), after the implementation of the VDO program, the mean total costs had a statistically significant level decrease by $283.76 (95% CI: -$409.06 to -$158.45; *p* < 0.01). There was also a significant decrease in the trend by $15.59 per month (95%: CI -$25.07 to -$6.11 per month; *p* < 0.01). In Model 2, after adjusting for age, gender and subsidy status, there was a statistical significant level decrease in the mean costs by $284.10 (95% CI: -425.08 to -143.12; *p* < 0.01), but the decrease in trend became statistically insignificant (-$12.21 per month, 95% CI: -$25.06—$0.65 per month; *p* = 0.07). Upon further adjustment for comorbidities and ASA score, level decrease remained statistically significant (-$327.23, 95% CI: -$421.04 to -$233.43; *p* < 0.01) and a statistically significant decreasing trend in cost was again observed (-$13.75 per month, 95%CI: -$23.19 to -$4.30 per month; *p* < 0.01) Results are summarised in Table 3.

**Table 3:** Interrupted time series models for level and trend changes for mean total cost before and after implementation of the VDO program

	**Model 1: Intervention**		**Model 2: Model 1 + age, gender and subsidy status**		**Model 3: Model 2 + CCI and ASA score**	
	**Estimates**	***p*-value**	**Estimates**	***p*-value**	**Estimates**	***p*-value**
**Intercept**	3110.10 (3035.73—3184.46)	< 0.01	4019.94 (1271.92—6767.98)	< 0.01	1270.57 (-1883.33—4424.47)	0.43
**Time**	-0.28 (-4.65—4.10)	0.90	-2.46 (-9.150—4.226)	0.47	0.60 (-5.65—6.86)	0.85
**Level**	-283.76** (-409.06—-158.45)	** < 0.01**	-284.10** (-425.08—-143.12)	** < 0.01**	-327.23** (-421.04—-233.43)	** < 0.01**
**Trend**	-15.59** (-25.07—-6.11)	** < 0.01**	-12.21 (-25.06—0.65)	0.07	-13.75** (-23.19—-4.30)	** < 0.01**
**Age**			-8.69 (-46.76—29.37)	0.66	-23.60 (-59.82—12.62)	0.21
**Gender**			-36.09 (-989.69—917.52)	0.94	-117.40 (-920.28—685.47)	0.78
**Subsidy Status**			-331.19 (-1142.24—479.85)	0.43	-266.69 (-989.59—456.22)	0.47
**CCI**					39.38 (-645.36—724.12)	0.91
**ASA Score**					1710.91** (1089.18—2332.64)	** < 0.01**

^*^p < 0.05, **p < 0.01

### Quality outcome score

Figure 1b showed the mean quality outcome score of cataract surgery from January 2015 to December 2018. In the months prior to the VDO program, the mean quality outcome score ranged from 9.82 to 9.96 and had an increasing trend. In Model 1 (unadjusted), after the implementation of the VDO program, the mean quality outcome score showed a statistically significant level increase of 0.015 (95% CI: 0.005 to 0.025; *p* < 0.01) but a small decreasing trend was noted (-0.002 per month, 95% CI: -0.002 to -0.001; *p* < 0.01). In Model 2, after adjusting for age, gender and subsidy status, the level increase in mean quality outcome score remained statistically significant (0.025, 95% CI: 0.012 to 0.038; *p* < 0.01) and the trend change became statistically insignificant (-0.002 per month, 95% CI: -0.003 to 0.000; *p* = 0.05). Further adjustment for comorbidities and ASA score in Model 3 had minimal impact on the level (0.028, 95% CI: 0.016 to 0.040; *p* < 0.01) and trend (-0.005 per month, 95% CI: -0.006 to 0.003; *p* = 0.38) changes. Results are summarised in Table 4.

**Table 4:** Interrupted time series models for level and trend changes for quality outcome score before and after implementation of the VDO program

	**Model 1: Intervention only**		**Model 2: Model 1 + age, gender and subsidy status**		**Model 3: Model 2 + CCI and ASA score**	
	**Estimates**	***p*-value**	**Estimates**	***p*-value**	**Estimates**	***p*-value**
**Intercept**	9.877 (9.872—9.882)	< 0.01	9.504 (9.074—9.933)	< 0.01	9.922 (9.439—10.404)	< 0.01
**Time**	0.001** **(0.001—0.002)**	** < 0.01**	0.001** **(0.000—0.002)**	** < 0.01**	0.004** **(0.003—0.005)**	** < 0.01**
**Level**	0.015** **(0.005—0.025)**	** < 0.01**	0.025** **(0.012—0.038)**	** < 0.01**	0.028** **(0.016—0.040)**	** < 0.01**
**Trend**	-0.002** **(-0.002—-0.001)**	** < 0.01**	-0.002 (-0.003—0.000)	0.05	-0.005 (-0.006—0.003)	0.38
**Age**			0.006 (0.000—0.012)	0.07	0.005* **(0.001—0.009)**	**0.02**
**Gender**			-0.038 (-0.201—0.126)	0.66	-0.082 (-0.242—0.079)	0.32
**Subsidy Status**			-0.016 (-0.158—0.126)	0.83	0.223 (0.051- 0.394)	0.015
**CCI**					-0.202** **(-0.305—-0.099)**	** < 0.01**
**ASA Score**					-0.197** **(-0.272—-0.121)**	** < 0.01**

^*^p < 0.05, **p < 0.01

### Sensitivity analysis

In many instances, especially in the healthcare setting, policy implementation may not be instantaneous. This often leads to a delay in program implementation. Hence, we considered a three-month phase-in period to test the robustness of our conclusions. We also included a separate analysis that included patients with multiple eye surgeries in the same admission, and we found that the findings from these analyses were similar.

## Discussion

The VDO program for cataract surgery introduced in NUH in June 2017 was associated with a reduction in cost without compromising on quality outcomes. Implementing a data reporting tool such as VDO can provide valuable information for physicians to improve the quality of care. Our study showed that after implementing the VDO program for cataract surgery, the total cost of cataract surgery significantly decreased in trend over time, while quality outcomes remained high. Our results are consistent with several studies evaluating VDO programs on various medical conditions. Studies done in the University of Utah [4, 17–20] used VDO as an information tool to improve quality outcomes and have identified cost drivers to reduce cost and improve value for patients. Lee et al. [4] evaluated the implementation of the VDO Program on three hospital processes, total hip and knee joint replacement, hospitalist laboratory utilisation, and management of sepsis. In their study, the physicians in charge of the speciality were given access to a tool with information about outcomes and costs and worked with quality improvement specialists. All three processes showed significant improvement in quality outcomes during the post VDO implementation year. Two studies from the same team [18, 19] used the VDO program to identify variability in cost for spinal procedures (Anterior Cervical Discectomy and Fusion [ACDF] and Lumbar Interbody Fusion). Karsy et al. [17] and Tashjian et al. [20] explored cost drivers to identify strategies to reduce cost in transsphenoidal resections of pituitary adenomas and outpatient arthroscopic rotator cuff repair surgery, respectively. These exploratory studies suggest that efforts to improve the value of such clinical procedures should target high-cost components such as facility costs and supplies and implant costs. The authors concluded that targeted programs such as decanting low-risk patients to step-down care facilities to reduce the length of stay or interventions to negotiate a reduction in supplies and implant cost can improve value in healthcare for such procedures.

Similarly, in our study, several initiatives informed by data from the VDO tool were implemented to improve the cost and outcomes of cataract surgery after VDO implementation. For example, there was an initiative to improve the workflow for patients undergoing surgery to reduce the time taken for patients to change into a surgical gown. This allows the surgery to be done more quickly and thus reduces cost. Sedation practices were also updated, which reduces the sedation patients require for the surgery. This allows them to be discharged earlier as they get out of the sedation post-operation more quickly. There were also several changes in administrative practices which aim to improve surgical outcomes. For elective surgeries, consultants can only book patients into their own surgery list. This ensures that they are more familiar with the patient’s medical condition and have at least seen them once in their clinic before operating. Bed occupancy practices were also changed where patients were allocated a chair instead of a bed if they are fit enough to recuperate on a recliner post operation. Room charges were consequently reduced for this group of patients. There was also an increase in the proportion of senior to junior doctors after VDO implementation. Such initiatives appear to have successfully reduced cost in patients undergoing cataract surgery, especially at the beginning of the intervention period where most of the administrative improvements take place.

However, our study only found minimal improvement in the quality outcome score. A plausible reason could be the ceiling effect in tracking the ten quality outcomes. The mean score for the quality outcome score is 9.91 out of 10, and about 94% of the episodes met all ten quality outcome measures. Hence, the room for improvement may be limited due to this ceiling effect. As a comparison, in the study by Lee et al. [4], only 54% of their total joint replacement cases met all their quality outcome measures before the implementation of their program. Therefore, the authors reported a 26% increase in cases meeting all their quality outcome measures one year after VDO program implementation. In order to overcome such ceiling effects and measure improvement in quality outcomes, a future study can attempt to incorporate more diverse quality outcome measures that are more sensitive and important to patients, such as health-related quality of life outcomes, pain measures, perspectives of the patients (e.g. using validated questionnaires such as the Catquest-9SF [21]), which takes into account the floor and ceiling effects during their validation process. These can be administered and be included as part of the quality outcome measures. Patients should also be consulted when formulating the outcome measures as ultimately, they are the ones deriving value out of the surgery.

Our results demonstrate an important lesson for public health. There is a possibility to increase value of healthcare if one measures, records, and makes decisions based on metrics important to patients. By providing physicians with data, we can reduce cost without compromising quality outcomes or even improve quality outcomes as demonstrated by some of the studies mentioned. There may be a benefit for physicians to understand costs and outcomes for individual patients with defined clinical indicators. The National Academy of Medicine [22] had also identified that healthcare delivery variability presents an excellent opportunity to improve value by improving quality and reducing cost. This can be done via process improvements or standardisation. With a simple data reporting tool such as the VDO program, the underlying factors of cost variability can be identified and serve as an essential starting point for targeted process improvements or standardisation. For example, in our case of cataract surgery, surgical services and room charges costs were the main cost drivers. Therefore, workflow improvements such as those described above were implemented to reduce costs.

For other clinical conditions, which may have high variability in facility fees [17–19], interventions around right-siting of care or defined clinical pathways may be helpful. Allowing quality outcome measures to be determined by physicians themselves permits the definition of clear goals and targets. It also promotes ownership and a sense of shared responsibility for quality improvement. The ability to track these quality and cost improvements of the interventions promptly allows greater physicians engagement and reinforce the positive initiatives. Healthcare providers in Singapore are also increasingly being held accountable for the quality and cost of care they provide. The recent appointment of the Fee Benchmarks Advisory Committee [23] and the Healthcare Services Act [24] serve as measures to maintain quality and ensure transparency of costs to allow patients to make informed choices. In addition to these oversights in place, the VDO program has the potential to reconciles both the cost and quality outcomes within a complete framework for value improvement.

Our study has several strengths. As described above, we use a solid quasi-experimental interrupted time series design which allows us to control for pre-existing levels and trends and detect any changes in outcomes as the VDO program was implemented. A time series model is also crucial as healthcare improvements usually take place over a period of time, which the model is able to capture, as compared to a pre-post study design which cannot detect trends. Adjusting for baseline trend using this method enables us to control for most threats to internal validity. Additionally, our model also included additional adjustments variables most likely to confound cost and quality outcomes. Hence, the results obtained are a more robust reflection of the effects of VDO implementation. The study also has adequate pre- and post-implementation timepoints, and a large sample size of cataract surgery episodes as it is one of the more commonly done procedures within NUH. This allows us to sufficiently power the ITS analysis [25]. Sensitivity analysis was also done using a 3-month phase-in period as well as a wider inclusion criteria. The consistent findings from the analyses give us greater confidence in the validity of our final model.

Our study also had several limitations. Firstly, the analysis was based on a single interrupted time series. This study design is unable to eliminate confounding due to co-interventions or other unrelated discontinuities occurring around the time of the VDO implementation. A way to reduce such potential confounding is to add a control group from other local hospitals without the VDO program, but this data was not available. Next, the data used in this analysis are based on individual cataract surgery episodes rather than individual patient-level data. This is due to limitations in VDO system architecture. A patient may be admitted for bilateral cataract surgery sequentially, and the VDO system will identify them as two separate episodes. A potential issue that can arise is that such episodes may be associated with one another if they are the same patient, thus violating the independence assumption of the linear model of the ITS analysis. However, it is unlikely that the proportion of such individuals will be significant due to the increased frequency of immediately sequential, bilateral cataract surgery done worldwide [26]. The current VDO system architecture has also been upgraded to be able to identify patient-level data, but as the study period is from 2015, our study is unable to obtain such data. Finally, nine out of ten quality outcome measures are clinical measurements. There is a disproportionate focus on clinical measurements for quality outcomes. The only patient-reported outcome measure (PROM) is the patient experience score. Future studies can include more PROMs, especially cataract surgery specific questionnaires such as the Catquest-9SF [21] or Cat-PROM5 [27]. The benefits of including such measures not only will enable a more holistic evaluation of outcomes but may also alleviate the ceiling effect for the quality outcome score that we have discussed earlier.

## Conclusion

In summary, the implementation of the VDO program is associated with reduction in cost, without compromising quality outcomes for patients undergoing cataract surgery in NUH. The VDO program has the potential to promote value-based healthcare by identifying cost drivers and catalysing the targeted cost and quality outcome improvement projects. However, further research and improvements to the VDO program and study design are needed to demonstrate the generalisability and scalability of the VDO program across other clinical conditions, departments, and healthcare systems.

## Data Availability

Available from corresponding author on reasonable request.
